# *Fusarium* Head Blight on Wheat: Biology, Modern Detection and Diagnosis and Integrated Disease Management

**DOI:** 10.3390/toxins15030192

**Published:** 2023-03-03

**Authors:** Elias Alisaac, Anne-Katrin Mahlein

**Affiliations:** 1 Institute of Crop Science and Resource Conservation (INRES), Plant Diseases and Plant Protection, University of Bonn, 53115 Bonn, Germany; 2 Institute for Grapevine Breeding, Julius Kühn-Institut, 76833 Siebeldingen, Germany; 3 Institute of Sugar Beet Research (IfZ), 37079 Goettingen, Germany

**Keywords:** wheat scab, mycotoxins, yield losses, epidemics, monitoring, decision making, control measures, resistance, remote sensing

## Abstract

*Fusarium* head blight (FHB) is a major threat for wheat production worldwide. Most reviews focus on *Fusarium graminearum* as a main causal agent of FHB. However, different *Fusarium* species are involved in this disease complex. These species differ in their geographic adaptation and mycotoxin profile. The incidence of FHB epidemics is highly correlated with weather conditions, especially rainy days with warm temperatures at anthesis and an abundance of primary inoculum. Yield losses due to the disease can reach up to 80% of the crop. This review summarizes the *Fusarium* species involved in the FHB disease complex with the corresponding mycotoxin profiles, disease cycle, diagnostic methods, the history of FHB epidemics, and the management strategy of the disease. In addition, it discusses the role of remote sensing technology in the integrated management of the disease. This technology can accelerate the phenotyping process in the breeding programs aiming at FHB-resistant varieties. Moreover, it can support the decision-making strategies to apply fungicides via monitoring and early detection of the diseases under field conditions. It can also be used for selective harvest to avoid mycotoxin-contaminated plots in the field.

## 1. Introduction

Wheat is an essential food around the world, in addition to its use as feed and fiber for livestock and energy production. It is the first crop in the world with a harvested area of 219 million hectares/year and a production of 760 million tons/year (average of 10 years). During the last decade, wheat yield reached 3475 kg/ha with an average increase of 605 kg/ha over the previous decade [1]. The genus wheat (*Triticum* L. 1753) is a member of the grass family *Poaceae* with different cultivated species, mainly the tetraploid durum wheat *Triticum durum* 2n = 28 with the genome set AABB and the hexaploid wheat *Triticum aestivum* 2n = 42 with the genome set AABBDD [2]. Wheat yield is affected by biotic (pests and pathogens) and abiotic stresses as a result of environmental conditions, stress occurrence, and genetic prevalence. Among the biotic stresses, pathogens (i.e., fungi, viruses, and bacteria) may contribute to average global losses of 21.5% of wheat yield [3].

*Fusarium* head blight (FHB) is one of the most relevant fungal diseases of wheat associated with different fungal species from the genus *Fusarium* [4,5]. FHB causes significant losses in wheat yield because the affected grains are small, shrunken, of low mass and quality, and contaminated with mycotoxins, which are harmful to humans and in animal nutrition [6]. The main mycotoxins produced by *Fusarium* species are trichothecenes, zearalenones, fumonisins, and the emerging toxins, i.e., beauvericin, enniatins, fusaproliferin, and moniliformin [7]. FHB, also known as *Fusarium* ear blight or scab, was first described in England by Smith (1884) [8] as a new disease of wheat, barley, and ryegrass, attributing the infection to the causal agents *Fusisporium culmorum*, *hordei* and *Lolii* Wm.G. Sm. It was recorded as an important disease in the USA at the end of the 19th century. By the beginning of the 20th century, FHB was known in wheat production regions worldwide [5]. In the 1990s, McMullen et al. [9] characterized it as a re-emerging disease due to the frequent epidemics on wheat in the USA and Canada from 1991 to 1996. They assigned this to the fundamental changes in agricultural practices, mainly reduced tillage. FHB is also known as a disease complex since more than one species from the genus *Fusarium* are involved in this disease in addition to two species from the genus *Microdochium* [10]. The main difference between them is that *Fusarium* species produce a wide spectrum of mycotoxins while *Microdochium* species do not produce mycotoxins [10].

Because of the high toxicity of *Fusarium* mycotoxins and the effect of *Fusarium* head blight on wheat yield, it is important to integrate plant protection practices such as crop rotation, resistant varieties, and cultural practices up to the application of fungicide within the management strategy. To safeguard wheat yield and to produce products with high quality especially, the application of fungicides is necessary under certain environmental conditions. To increase knowledge about the relevant parameters of the epidemiology and to perform knowledge-based plant protection measures, it is important to investigate and develop new methods to predict and detect FHB epidemics early on cereals [11]. This is essential, for example, for making the decision to apply fungicides at a suitable time of infection.

Besides direct control, a highly effective strategy to control FHB is breeding varieties with appropriate resistance against this disease complex. Effective cultivar development needs interdisciplinary research, integrating plant breeding, phytopathology, and informatics. It also needs precise and innovative methods for identifying and characterizing disease symptoms at an early stage of pathogenicity [12]. Because conventional characterization of host plant genotypes is laborious, time-consuming and cost-intensive, this is a limiting factor in plant-breeding programs. Here, proximal sensing with optical sensors is a promising characterizing method. Various sensors are suitable for the detection, identification, and quantification of plant diseases, such as thermography, fluorescence, and spectral sensors [13]. Recently, hyperspectral imaging showed efficiency as a precise and non-destructive tool in characterizing the interaction of *Fusarium* spp. and wheat plants. It was efficiently used in quantifying wheat resistance to FHB [14], the assessment of *Fusarium* infection and mycotoxin contamination of wheat kernels and flour [15], and, combined with thermography and chlorophyll fluorescence, for the early detection and monitoring of FHB development on wheat [16].

Most available reviews focus on *Fusarium graminearum* as the main causal agent of FHB on wheat; therefore, this review summarizes the *Fusarium* species involved in the FHB disease complex with the corresponding mycotoxin profiles, disease cycle, and diagnostic methods. In addition, it displays the history of FHB epidemics, and the management strategy of the disease. Moreover, it discusses the role of remote sensing technology in diagnosing and phenotyping the disease symptoms on wheat spikes.

## 2. *Fusarium* Head Blight Epidemics on Wheat

The frequency of FHB epidemics has increased in the last decades due to the changes in agricultural practices, mainly the zero tillage regime in wheat fields, in addition to the increase in the area of wheat cultivation and the frequency of wheat in the crop rotation [17,18]. The incidence of FHB epidemics is highly correlated with weather conditions especially rainy days with warm temperatures at anthesis and an abundance of primary inoculum [17,18,19,20,21]. Several studies showed that FHB epidemics on wheat take place sporadically. In Europe, yield losses caused by FHB epidemics ranged between 40 and 50% in Romania and Hungary during the 1970s–1980s [5]. A recent study showed an increase in *Fusarium* mycotoxin contamination in European wheat during the years 2010–2019; however, there is a lack of data on FHB incidence in Europe [22]. Seven severe epidemics and 14 medium epidemics occurred during the second half of the last century in China. In 2012, more than 9.9 million hectares were affected in the main producing areas in China [23]. In Canada, FHB epidemics were recorded during the 1940s and 1980s [24]. In the last two decades, epidemics were reported in 11 out of 17 surveyed years with a disease severity of 1.5–57.8% [25]. In the 20th century, five severe epidemics were described in the USA from the 1910s to the 1930s. However, FHB re-emerged from 1991 to 1996 and caused a yield drop of up to 25% and economic losses of USD 1 billion [9]. It continued to occur frequently from 1997 to 2010 in several states in the USA, resulting in annual losses of up to 54.2% in 2003 in different states [6]. In Latin America, seventeen epidemics have been reported in Argentina from 1960 to 2012 with losses of up to 70% in some years [26]. Furthermore, a model-based assessment study analyzed the weather data from 1957 to 2006 in Brazil. It showed an increased FHB-risk index during the 1960s and higher frequency of high-risk years starting from 1990 [27]. Information about FHB epidemics in Australia are rare due to their sporadic nature. However, a severe epidemic was reported in 2010 with disease of 79% in some fields [20].

## 3. The Pathogen

### 3.1. Pathogen Taxonomy

**Figure 1:**
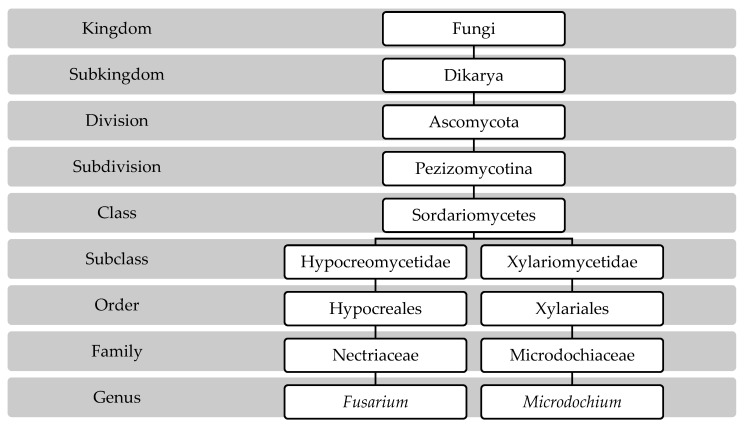
Taxonomical position of the genus *Fusarium* and *Microdochium* according to MycoBank database, 2022.

Since 2013, and after the changes in the International Code of Nomenclature for fungi, the name *Fusarium* was recognized as a unique name for all species including teleomorphs, which means that the name *Gibberella* and other names are not accepted anymore to indicate the sexual stage of these pathogens [28]. In addition, the subspecies of the genus *Microdochium* were elevated to species in 2005 and have become the accepted taxonomy. The following scheme shows the taxonomical position of the genus *Fusarium* and *Microdochium* according to the MycoBank database, 2022 [29] (Figure 1).

### 3.2. Fusarium Species Involved in FHB

*Fusarium graminearum* is the main pathogen of FHB worldwide [4,6,21,30]. However, different studies showed that other *Fusarium* species may contribute significantly to this disease in different areas of the world with different climate conditions. For example, *F. graminearum*, *F. culmorum*, *F. avenaceum*, *F. poae*, *F. tricinctum*, and *M. majus* were the dominant species in Europe [31,32,33]. In Canada, *F. avenaceum*, *F. equiseti*, *F. graminearum*, *F. poae*, and *F. sporotrichioides* were the most frequent species during the last two decades [25]. Alkadri et al. [34] recovered different *Fusarium* species from wheat in Syria. Table 1 shows the *Fusarium* species involved in FHB.

### 3.3. Life Cycle and Pathogenesis

**Figure 2:**
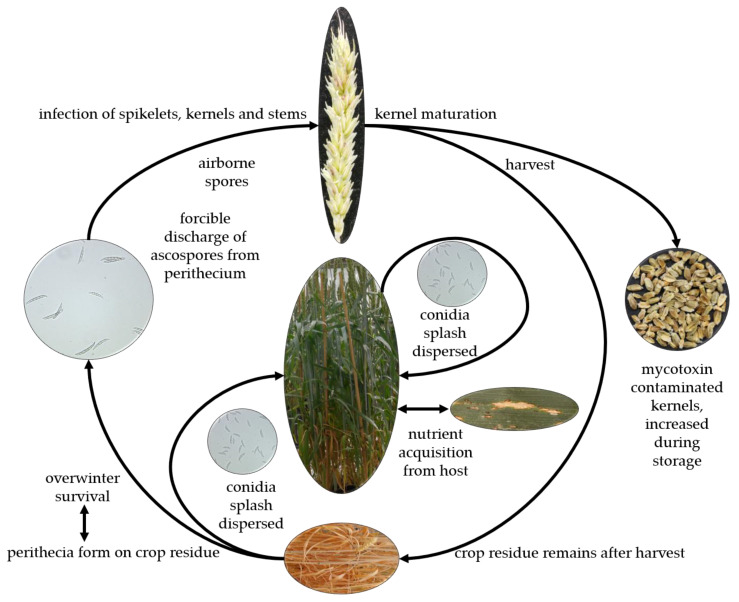
Disease cycle and symptoms of *Fusarium* head blight on wheat spikes and kernels.

*Fusarium* head blight is a monocyclic disease (Figure 2). The pathogen survives in the debris of the previous crop as macroconidia or within sexual structures called perithecia as ascospores; or as asexual spores called macroconidia or microconidia for the species that have only anamorph stage. These spores are considered the primary inoculum of the disease. In addition, gramineous and non-gramineous weeds are not only a host range of *Fusarium* but they can also serve as an alternative host and source of inoculum. At wheat anthesis, which is the susceptible stage for infection, and in favorable weather conditions, the inoculum is blown by the wind or splashed by rain and lands on open spikelets. On the spikelet tissue, the spores germinate and produce germination tubes [6,21,35,36,37,38]. After germination, the fungal hyphae spread on the surface of the ovary, palea, and lemma and start to produce mycotoxins without penetrating the spikelet tissue. Thereafter, the pathogen penetrates the host tissue, starting a biotrophic infection with an intercellular growth in the spikelet and turns to the necrotrophic stage with inter- and intracellular growth laterally and vertically within the spike (Figure 2). During this stage of pathogenicity, mycotoxins accumulate in the spike tissue as well as in the kernels, reducing the crop yield and quality [39,40,41].

### 3.4. Symptoms

The first symptoms appear as water-soaked spots on the infected spikelets; these symptoms develop to necrosis and in an advanced stage, the infected spikelets become bleached (Figure 2). The bleaching spreads to cover the entire spike resulting in premature white wheat heads. Under warm and humid weather conditions, pinkish-red mycelium appears on the infected tissue. The kernels resulting from the infected spikes are known as tombstones because they are light in weight, shriveled, discolored with a pinkish or chalky appearance, and poor quality (Figure 2) [6,9,35,42,43]. However, some less virulent pathogens, such as *F. poae,* may cause infection and result in high levels of mycotoxin contamination in the infected kernels without detectable symptoms on the spikelets or the spike [44,45].

**Table 1:** *Fusarium* and *Microdochium* species involved in *Fusarium* head blight on wheat with their teleomorph and mycotoxin profile.

Pathogen	Teleomorph	Mycotoxin Profile																			Reference
		Trichothecenes												Zearalenone	Fusaric Acid	Fumonisins	Emerging Toxins				
		Trichothecenes A							Trichothecenes B								Enniatins	Beauvericin	Fusaproliferin	Moniliformin	
		T-2 toxin	HT-2 toxin	DAS	MAS	NEO	NX-2	NX-3	DON	3-ADON	15-ADON	NIV	4-ANIV								
*F. acuminatum* Ellis & Everh.	+	+				+			+				+				+			+	[46,47,48]
*F. avenaceum* (Fr.) Sacc.	+																+	+		+	[46,47,49,50]
*F. crookwellense* L.W. Burgess, P.E. Nelson & Toussoun synonym *F. cerealis* (Cooke) Sacc.	-											+	+	+							[47,50,51,52]
*F. culmorum* (W.G. Sm.) Sacc.	-								+	+	+	+	+	+							[34,39,53,54,55,56]
*F. equiseti* (Corda) Sacc.	+			+		+			+			+	+	+			+				[34,48,57]
*F. graminearum* Schwabe	+						+	+	+	+	+	+	+	+							[34,46,53,56,58,59,60]
*F. lateritium* Nees	+																+				[61,62]
*F. oxysporum* Schltdl.	-														+					+	[7,63]
*F. poae* (Peck) Wollenw.	-	+	+	+	+							+	+				+	+			[7,46,49,57,64]
*F. proliferatum* (Matsush.) Nirenberg	+														+	+	+	+	+	+	[49,65,66]
*F. sambucinum* Fuckel	+	+	+	+	+	+						+					+	+		+	[49,61,67]
*F. semitectum* Berk. & Ravenel	-											+		+							[68,69]
*F. sporotrichioides* Sherb.	-	+	+	+	+	+						+		+			+	+			[49,57,70]
*F. subglutinans* (Wollenw. & Reinking) P.E. Nelson, Toussoun & Marasas	+																+	+	+	+	[7,61,71]
*F. tricinctum* (Corda) Sacc.	+																+			+	[49]
*F. verticillioides* (Sacc.) Nirenberg synonym *F. moniliforme* J. Sheld.	+															+				+	[7,72]
*Microdochium nivale* (Fr.) Samuels & I.C. Hallett	+																				[10]
*Microdochium majus* (Wollenw.) Glynn & S.G.Edwards	+																				[10]

Diacetoxyscirpenol (DAS); monoacetoxyscirpenol (MAS); neosolaniol (NEO); deoxynivalenol (DON); 3-acetyl-deoxynivalenol (3-ADON); 15-acetyl-deoxynivalenol (15-ADON); nivalenol (NIV); 4-acetyl-nivalenol (4-ANIV); zearalenone (ZEA); fusaric acid (FA); fumonisins (FUMs); enniatins (ENs); beauvericin (BEA); fusaproliferin (FP); moniliformin (MON).

## 4. Mycotoxins

**Figure 3:**
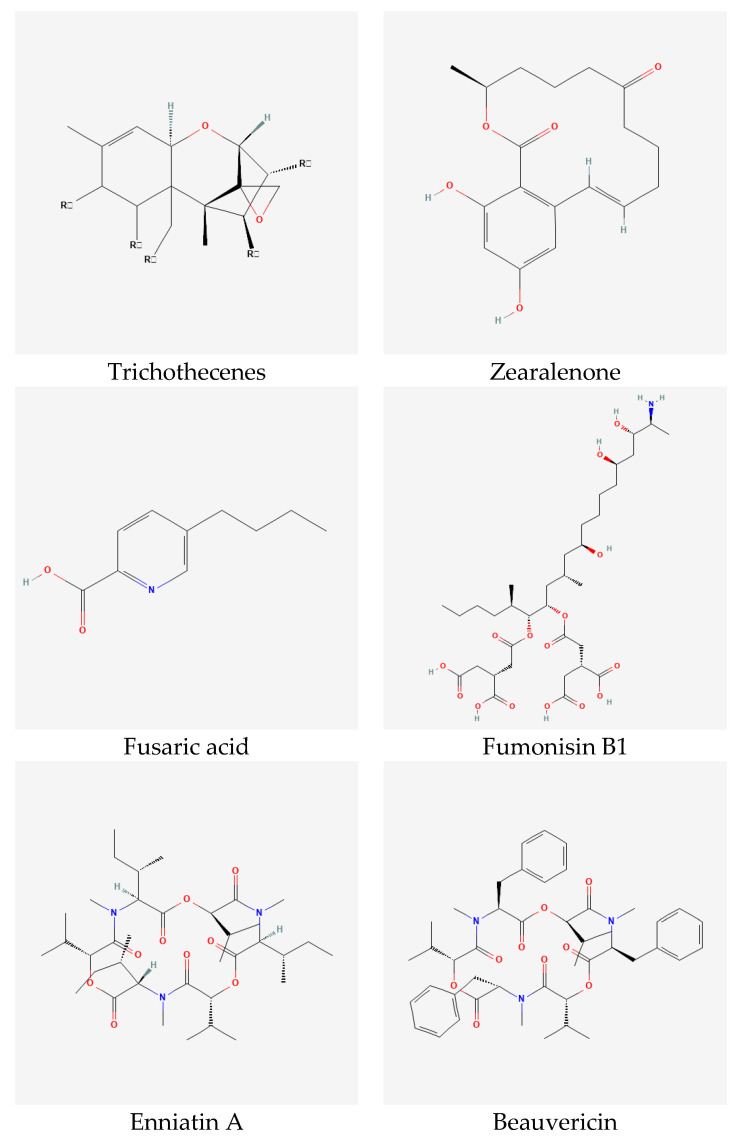

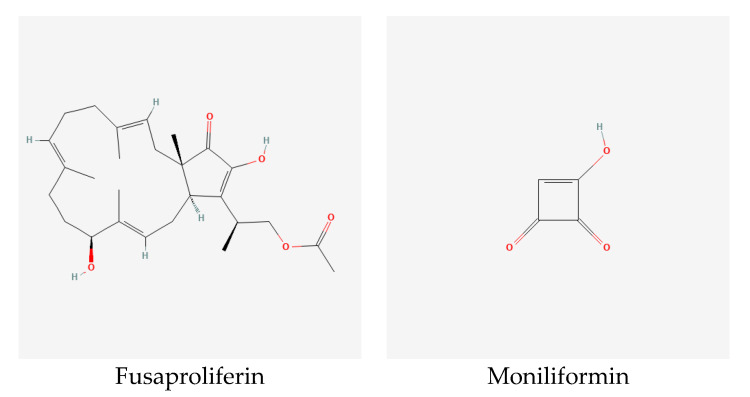
Chemical structure of the main mycotoxins produced by *Fusarium* species on wheat.

Mycotoxins are secondary metabolites produced by mold fungi such as *Alternaria*, *Aspergillus*, *Claviceps, Fusarium*, and *Penicillium*. *Fusarium* species involved in FHB produce a wide range of mycotoxins, mainly trichothecenes, zearalenone, fusaric acid, fumonisins, and emerging toxins, i.e., enniatins, beauvericin, moniliformin, and fusaproliferin (Figure 3). Due to the toxic effect of these metabolites on human and animal health, it is important to detect and quantify these toxins in food and feed. However, this process is challenging because it is expensive, time-consuming and laborious [15].

### 4.1. Trichothecenes

Trichothecenes are the most dominant group of *Fusarium* mycotoxins accompanying FHB infection on wheat worldwide [73]. This group is split, based on its chemical structure, into four subgroups A, B, C, and D [74]. However, trichothecenes produced by *Fusarium* spp. are A and B. The main difference between these two groups is the presence of ketone (=O) at C_8_ of trichothecenes backbone in trichothecenes B while it is absent in trichothecenes A [73]. In general, trichothecenes A are more toxic *in Animalia* compared with trichothecenes B; however, *in Planta*, trichothecenes B are more toxic [75]. Trichothecenes A include T-2 toxin, HT-2 toxin, diacetoxyscirpenol (DAS), monoacetoxyscirpenol (MAS), neosolaniol (NEO), NX-2 and NX-3. This group is mainly produced by *F. acuminatum*, *F. equiseti*, *F. graminearum*, *F. poae*, *F. sambucinum*, and *F. sporotrichioides*. Trichothecenes B include nivalenol (NIV), 4-acetyl-nivalenol (4-ANIV), deoxynivalenol (DON), 3-acetyl-deoxynivalenol (3-ADON) and 15-acetyl-deoxynivalenol (15-ADON). *Fusarium* species that produce trichothecenes B are *F. acuminatum*, *F. crookwellense*, *F. culmorum*, *F. equiseti*, *F. graminearum*, *F. poae*, *F. sambucinum*, *F. semitectum*, and *F. sporotrichioides* (Table 1). Trichothecenes B are known as a virulence factor of *Fusarium* spp. against wheat [76,77]. However, DON is more poisonous *in Planta* while NIV is more poisonous *in Animalia* [7]. The toxic effect of trichothecenes is the inhibition of protein synthesis in eukaryote by binding with 60S ribosomes [73,74,75].

### 4.2. Zearalenone

Zearalenone is also one of the dominant *Fusarium* mycotoxins on wheat worldwide; it is normally found in the same climate regions as trichothecenes [78,79,80,81]. Zearalenone derivatives, mainly, zearalanone, α- and β-zearalenol, and α- and β-zearalanol could be naturally produced by *Fusarium* spp. [7]. The main difference is the presence of ketone (=O) at C_12_ in zearalenone and zearalanone while it is hydroxyl (-OH) in α- and β- derivatives [82]. Zearalenone is of low acute toxicity either *in Planta* or *in Animalia* compared with trichothecenes [7,83]. *Fusaria* involved in zearalenone production are *F. crookwellense*, *F. culmorum*, *F. equiseti*, *F. graminearum*, *F. semitectum*, and *F. sporotrichioides* (Table 1). *In Animalia*, zearalenone has an estrogenic effect by binding to estrogen receptors which affect the sexual activities of animals [84].

### 4.3. Fusaric Acid

Fusaric acid is one of the first identified *Fusarium* mycotoxins; it is produced by a wide range of *Fusarium* species, and interestingly, by both trichothecene and fumonisin producers [63]. It is considered as a virulence factor for different *Fusaria*. Fusaric acid has virulent toxicity *in Planta*; however, its toxicity *in Animalia* is low to moderate. The toxic effects of fusaric acid include modifying the potential of the cell membrane and inhibiting ATP synthesis [85].

### 4.4. Fumonisins

Fumonisins were first identified in South Africa in 1988. They can be found mainly in maize products in regions with warm conditions. However, they can also be detected in other cereals especially wheat, barley and sorghum, and other plants such as soybean, asparagus, tea, and medicinal plants [86,87]. Fumonisins are polyketide hydrophilic mycotoxins and they contain a large number of derivatives. Therefore, they are classified in four main groups A, B, C, and P. Fumonisins B is the most widespread group and it contains FB1 which is of high concern regarding human and animal toxicity [7,87,88]. Exposure to fumonisins causes esophageal cancer and embryonal neural-tube defects in humans, leuko-encephalomalacia in equine and pulmonary edema in pigs [87,88,89]. A large number of *Fusarium* species are involved in fumonisin production; however, *F. verticillioides* and *F. proliferatum* are the main producers of these toxins [88].

### 4.5. Emerging Toxins

The emerging toxins of *Fusarium* are enniatins, beauvericin, fusaproliferin, and moniliformin [61]. The presence of emerging toxins is accompanied by traditional *Fusarium* toxins in cereals, particularly maize, wheat, barley, and oat, worldwide [62,67,71,90,91].

#### 4.5.1. Enniatins and Beauvericin

Enniatins and beauvericin are cyclohexadepsipeptides with a lipophilic nature [61]. The main chemical derivatives of enniatins that can be detected in cereals are ENA, ENA1, ENB, and ENB1 [92]. Besides their antibacterial, antifungal, insecticidal activities [92], they showed cytotoxic effect for different cell cultures *in vitro*; however, toxicity *in vivo* is limited to poultry especially in the liver [67,93]. In addition, they exhibited cytotoxicity against cancer cell lines suggesting them as pharmacological candidates to fight cancer [94]. Enniatins and beauvericin are produced by a wide spectrum of *Fusarium* species [67]; however, on wheat, they were recorded for *F. acuminatum*, *F. avenaceum*, *F. equiseti*, *F. lateritium*, *F. poae*, *F. proliferatum*, *F. sambucinum*, *F. sporotrichioides*, *F. subglutinans*, and *F. tricinctum* (Table 1).

#### 4.5.2. Fusaproliferin

Fusaproliferin is a bicyclic sesterterpene, which was later discovered in 1993 from *F. proliferatum* isolate. It can be produced simultaneously with a deacetylated form in a 3:1 ratio [61]. Fusaproliferin shows toxicity on insect and mammalian cells in addition to poultry embryos [7]. On wheat, fusaproliferin production was reported for *F. proliferatum* and *F. subglutinans* (Table 1).

#### 4.5.3. Moniliformin

Moniliformin is a small molecule with high polarity; it can be found in nature as a sodium or potassium salt. Moniliformin was first identified as a mycotoxin of *F. moniliforme* that was renamed *F. verticillioides* [61]. The toxic effect of moniliformin is by disrupting thiamine enzymes that affect cellular energy supply. This leads to acute heart failure, pulmonary and immunity disruption in animals [67]. On wheat, moniliformin accompanied FHB infected with *F. acuminatum*, *F. avenaceum*, *F. oxysporum*, *F. proliferatum*, *F. sambucinum*, *F. subglutinans*, *F. tricinctum*, and *F. verticillioides* (Table 1).

### 4.6. Masked Mycotoxins

The term “masked mycotoxin” indicates a mycotoxin biologically modified by a conjugation reaction from the plants as a detoxification mechanism. This was suggested to differentiate them from other types of biological modification of mycotoxins, e.g., by animals, fungi, and microbiota of animals and humans. In addition, to discriminate them from chemically modified mycotoxins (i.e., thermally and non-thermally) and matrix-associated mycotoxins [95]. The main concern regarding masked mycotoxins is that they are not detectable by traditional analysis, and they are hydrolyzed through digestion into their parental mycotoxins or even more or less toxic compounds [95]. Different masked mycotoxins were reported in cereal grains. DON-3-glucoside (DON-3-G), NIV-3-glucoside (NIV-3-G), 4-ANIV-glucoside (4-ANIV-G), T-2-3-glucoside (T-2-3-G), HT-2-3-glucoside (HT-2-3-G), and ZEA-14-glucoside (ZEA-14-G) were reported to occur in wheat [96]. In their original situation, the toxicity of masked trichothecenes and zearalenone was shown to be significantly lower than their free parental toxins. They also showed stability in the upper gastro-intestinal tract. However, the main danger of these toxins is that they are hydrolyzed by the intestinal microbiota after ingestion [97,98]. Therefore, these masked mycotoxins must be legislated and controlled in the food and feed chain [53].

## 5. *Fusarium* Head Blight Diagnosis on Wheat

There are different methods to diagnose the fungal pathogens involved in FHB on wheat. The classical method is pathogen re-isolation on selective media and identifying the fungus based on the morphological characteristics of the spores or the colony. The immunological method uses specific antibodies against a specific protein or protein complex produced by the fungus. However, the most specific method is the molecular method using specific primers that target a specific region in the DNA of the fungus.

### 5.1. Selective Media

*Fusarium* species can be diagnosed based on the visual and microscopical characteristics of the colony and the spores after re-isolating the fungus on selective medium. Different media showed selectivity to *Fusarium* spp., e.g., Czapek Dox iprodione dichloran agar (CZID), dichloran-chloramphenicol peptone agar (DCPA), malachite green agar (MGA 2.5), modified Czapek Dox agar (MCz), Nash and Snyder medium (NS), and potato dextrose iprodione dichloran agar (PDID). However, MGA 2.5 was recommended as a selective medium for *Fusarium* re-isolation from naturally infected kernels [99]. Furthermore, differentiation between *Fusarium* species was possible based on their pigmentation on CZID [100]. Recently, different media containing the bacterial toxin “toxoflavin” produced by the *Burkholderia glumae* showed selectivity to *Fusarium* species [101]. However, this method is laborious and time-consuming and it needs experts in fungal taxonomy to diagnose the disease at the species scale.

### 5.2. Immunological Method

Enzyme-linked immunosorbent assay (ELISA) is used as a diagnostic method for *Fusarium* using poly- or monoclonal antibodies. These antibodies are obtained after immunization of animals or cell lines by exoantigens secreted by *Fusarium* [102,103]. However, the main drawback of this method is that it is genus specific [104].

### 5.3. Molecular Method

The polymerase chain reaction (PCR) was invented in 1984 and became widely used in plant pathogen detection and quantification with high sensitivity and specificity [105,106]. The PCR costs reduced with the introduction of the DNA polymerase (Taq) of the high-temperature tolerant bacteria *Thermus aquaticus* in 1988. This allowed automated thermal cycling and abolished the need for enzyme refreshment after each cycle [105]. The PCR allows the detection of plant diseases before the symptoms become visible. Moreover, it differentiates between fungal species scale even when they have morphological similarities. Different genomic regions are used to design species-specific primers, e.g., internal transcribed spacers (ITS), intergenic spacer (IGS) regions, and protein-coding genes [107]. Different primers were developed to detect *Fusarium* species involved in FHB (Table 2). However, primers targeting *F. lateritium* and *F. semitectum* showed cross-hybridization with other *Fusarium* species [108].

**Table 2:** Forward and reverse primers sequences used to amplify specific fragments of fungal DNA of *Fusarium* species.

Pathogen	Primer Name	Primer Sequence (5′-3′)	Amplified Fragment	Reference
*F. acuminatum*	FAC-F FAC-R	GGGATATCGGGCCTCA GGGATATCGGCAAGATCG	602 bp	[109]
*F. avenaceum*	Fave574 fwd Fave627 rev	TATGTTGTCACTGTCTCACACCACC AGAGGGATGTTAGCATGATGAAG	EF1α gene	[110]
*F. crookwellense* synonym *F. cerealis*	CRO-A F CRO-A R	CTCAGTGTCCACCGCGTTGCGTAG CTCAGTGTCCCAATCAAATAGTCC	842 bp	[111]
*F. culmorum*	OPT18 F OPT18 R	GATGCCAGACCAAGACGAAG GATGCCAGACGCACTAAGAT	472 bp	[112]
*F. equiseti*	Feq-F Feq-R	GGCCTGCCGATGCGTC CGATACTGAAACCGACCTC	990 bp	[113]
*F. graminearum*	Fg16N F Fg16N R	ACAGATGACAAGATTCAGGCACA TTCTTTGACATCTGTTCAACCCA	280 bp	[114]
*F. lateritium*				
*F. oxysporum*	FOF1 FOR1	ACATACCACTTGTTGCCTCG CGCCAATCAATTTGAGGAACG	340 bp	[115]
*F. poae*	Fp82 F Fp82 R	CAAGCAAACAGGCTCTTCACC TGTTCCACCTCAGTGACAGGTT	220 bp	[116]
*F. proliferatum*	Fp3-F Fp4-R	CGGCCACCAGAGGATGTG CAACACGAATCGCTTCCTGAC	230 bp	[117]
*F. sambucinum*	FSF1 FSR1	ACATACCTTTATGTTGCCTCG GGAGTGTCAGACGACAGCT	315 bp	[115]
*F. semitectum*				
*F. sporotrichioides*	Fspor F1 Lanspo R1	CGCACAACGCAAACTCATC TACAAGAAGACGTGGCGATAT	332 bp	[118]
*F. subglutinans*	61-2F 61-2R	GGCCACTCAAGAGGCGAAAG GTCAGACCAGAGCAATGGGC	445 bp	[119]
*F. tricinctum*	Ftri573 fwd Ftri630 rev	TTGGTATGTTGTCACTGTCTCACACTAT TGACAGAGATGTTAGCATGATGCA	EF1α gene	[110]
*F. verticillioides* synonym *F. moniliforme*	VERT1 VERT2	GTCAGAATCCATGCCAGAACG CACCCGCAGCAATCCATCAG	800 bp	[120]
*Microdochium nivale*	Y13N F Y13N R	ACCAGCCGATTTGTGGTTATG GGTCACGAGGCAGAGTTCG	300 bp	[121]
*Microdochium majus*	Y13M F Y13M R	CTTGAGGCGGAAGATCGC ATCCCTTTTCCGGGGTTG	220 bp	[121]

## 6. Integrated Management of *Fusarium* Head Blight

The effective management of FHB is challenging due to several factors. Firstly, maize intensification and reduced tillage increased the frequency of FHB epidemics during the last decades. This is because maize is the main host of *Fusarium* species, which serves as a source of the inoculum, and reduced tillage helps to keep this source available during wheat vegetation. In addition, wheat comes very often after maize in the crop rotation, which increases the disease incidence during the availability of the inoculum. Secondly, the visible FHB symptoms appear on wheat spikes at a later stage of pathogenicity, and during this stage, it is too late for fungicide application because the kernels have been contaminated with *Fusarium* mycotoxins. In addition, traditional disease control using fungicides involves different disadvantages mainly costs, bio- and eco-hazards, relatively short lifetime due to fungicide resistance, and low availability for smallholder farmers. Moreover, the environment and health protection measures lead to continuous regulatory changes regarding the availability and applicability of fungicides [122]. This shows the need for an integrated management strategy that incorporates cultural practices, resistant varieties, and bio- and chemical measures to control the disease.

### 6.1. Cultural Practices

Adopting moderately resistant varieties combined with variety rotation and using varieties with different maturities in addition to spreading anthesis times by disseminating planting dates showed efficiency in FHB control [123,124]. In addition, the reduction of inoculum pressure during wheat anthesis can play a significant role in disease management. This can be achieved by plowing the soil and burying the residues of the previous crop especially if this crop is one of the main hosts of *Fusarium* species, such as maize and barley. This practice prohibits perithecia formation and ascospore discharge during wheat spike development [21,125]. Another practice to reduce inoculum pressure is avoiding FHB cultural hosts, e.g., maize as a previous crop in wheat fields [126].

### 6.2. Host Plant Resistance to Fusarium Head Blight

Components of wheat resistance to FHB include passive resistance represented by morphological and phenological features and active resistance represented by physiological features [127]. Morphological and phenological features that are involved in passive resistance are plant height, wheat awns, narrow and short floral opening, and the time of retained anthers. Plant height: tallness helps wheat spikes to stand away from splashed rain droplets that carry the inoculum from the soil surface and crop residues. Wheat awns: awns trap the inoculum and increase natural infection while their absence reduces it [127]. A narrow and short floral opening reduces the floret’s exposure to the inoculum and increases resistance while retained anthers and pollen might trap the inoculum and catalyze spore germination and fungal penetration [128]. Active resistance can be classified into the following types: resistance to initial penetration or infection (Type I resistance), resistance to fungal spread within the spike from the infected spikelet (Type II resistance) [129], resistance to kernel infection and tolerance against FHB (Types III and IV, respectively) [130], and resistance to trichothecenes (Type V) [73].

Wheat resistance to FHB is a quantitative trait, which means that many genes with cumulative effects are involved in this trait. Environmental conditions have a significant effect on this trait resulting in various resistance levels in different environments [128]. Durum wheat is known to be highly susceptible to FHB due to the scarcity of resistance sources in the tetraploid gene pool [30]. To date, seven quantitative trait loci (QTLs) were officially given gene names, most of them from Chinese hexaploid wheat (Table 3) [131]. However, the direct introgression of these sources in breeding programs is still difficult due to undesirable agronomic traits [131]. Steiner et al. [128] suggested integrating genomic selection based on genome-wide prediction models with marker-assisted selection for QTL and classical phenotypic selection based on visible symptoms in breeding programs for FHB resistance.

**Table 3:** QTLs involved in wheat resistance to *Fusarium* head blight.

QTL	Location	Source	Resistance Type	Reference
*Fhb1*	3BS	Sumai 3 and Nyubai	Type II	[132]
*Fhb2*	6BS	Sumai 3	Type II	[133]
*Fhb3*	7AS	*Leymus racemosus*	Type II	[134]
*Fhb4*	4BL	Wangshuibai	Type I	[135]
*Fhb5*	5AS	Wangshuibai and Sumai 3	Type I	[136]
*Fhb6*	1AS	*Elymus tsukushiensis*	Type II	[137]
*Fhb7*	7D	*Thinopyrum ponticum*	Type II	[138]

### 6.3. Biological Control

Biological control uses microorganisms antagonistic to *Fusarium* species or biological secondary metabolites to control FHB on wheat. These microorganisms can be applied to the residues of the previous crop to inhibit perithecia development or directly to wheat spikes. For example, bacteria from *Bacillus* spp., *Lysobacter enzymogenes, Pseudomonas* spp. and *Streptomyces* spp., and fungi from *Aureobasidium pullulans*, *Clonostachys rosea*, and *Trichoderma* spp. showed effectivity against *Fusarium* [139]. The fungus *Clonostachys rosea* was applied on wheat residues infected with different *Fusarium* species under field conditions. *Fusarium* growth measured as fungal DNA reduced between 68 and 98% after 90 days of treatment and was undetectable after 180 days [140]. Comby et al. [141] reported three new fungal species, namely *Aureobasidium proteae*, *Phoma glomerate*, and *Sarocladium kiliense,* with a high protection ratio between 75 and 100% on detached wheat spikelets. The basidiomycetous yeast *Cryptococcus nodaensis* OH 182.9 was isolated from wheat anthers [142]. This isolate presented reduced disease severity of between 45 and 60% under controlled and field conditions [143,144]. Zhang et al. [145] isolated 113 endophytes from roots, stems, leaves, and spikelets of wheat and tested their antagonistic effect against *F. graminearum* on detached wheat spikes. Six isolates were shown to inhibit *F. graminearum* growth while the strain XS-2 of *Bacillus amyloliquefaciens* reduced disease severity on detached wheat spikes significantly. *In vitro*, *B. subtilis* SG6 inhibited *F. graminearum* growth, sporulation, and DON concentration with ratios of 88, 96, and 100%, respectively, while in the field, the same strain significantly reduced disease incidence, FHB index, and kernel DON contamination when it was applied from anthesis until soft dough [146]. The usage of biochemical compounds proved to be effective in FHB control. Chitosan (the deacetylated derivative of chitin) inhibited the fungal growth and DON contamination in irradiated wheat kernels [147]. It also reduced disease severity and DON contamination by ≥74% under greenhouse and field conditions [148]. Drakopoulos et al. [149] tested botanical aqueous extracts of white mustard (*Sinapis alba*) and Chinese galls (*Rhus chinensis*) against *F. graminearum in vitro*. All these compounds fully inhibited mycelium growth, conidial, and ascospore germination. Moreover, they reduced perithecia formation and ascospore discharge up to 50 and 6%, respectively.

### 6.4. Chemical Control

Effective chemical control of FHB should be combined with other management practices [125,150]. The critical time for fungicide application is the susceptible stage, i.e., anthesis stage and 10 days after anthesis. However, a limited application period, anthesis heterogenicity, and weather conditions at this stage might be challenging for effective fungicide application; this may require multiple applications to achieve efficient disease control [139,151]. Demethylation inhibitor (DMI) fungicides, namely metconazole, prothioconazole, tebuconazole, prothioconazole + tebuconazole were shown to be more effective than propiconazole [6,152]. Another factor that affects fungicide efficiency is spike coverage during application, which is affected by the nozzle type, and spray angle. Lehoczki-Krsjaket al. [153] showed that two sideward-spraying (90 and 120° for forward and backward streams, respectively) increased fungicide content between 1.08 and 1.43 times in wheat spikes compared with vertical spraying. Moreover, increasing spike coverage from 19 to 37% reduced FHB incidence and DON content significantly for all tested fungicides [154].

### 6.5. Predicting and Detecting Disease Incidence

One of the prediction practices is risk assessment using disease prediction models based on weather conditions and the history of FHB epidemics in the growing region [6]. In addition, the effective monitoring of disease incidence in the field helps in early disease detection and supports the decision-making strategy to apply fungicides at a suitable time. Experts and prognosis models are based on information about the dominant *Fusarium* species, inoculum availability, resistance degree of the cultivated wheat variety, anthesis period, and the previous crops in the surrounding area. In addition, information about favorable weather conditions (e.g., rainfall, temperature, and relative humidity) for FHB incidence during wheat vegetation are required as input data for these models. These two practices could be effective tools to prevent quantity and quality losses in wheat yield caused by FHB. These models can be supported or improved by innovative digital technology and remote sensing data to realize knowledge-based plant protection management.

## 7. Remote Sensing for Monitoring and Phenotyping *Fusarium* Head Blight

**Figure 4:**
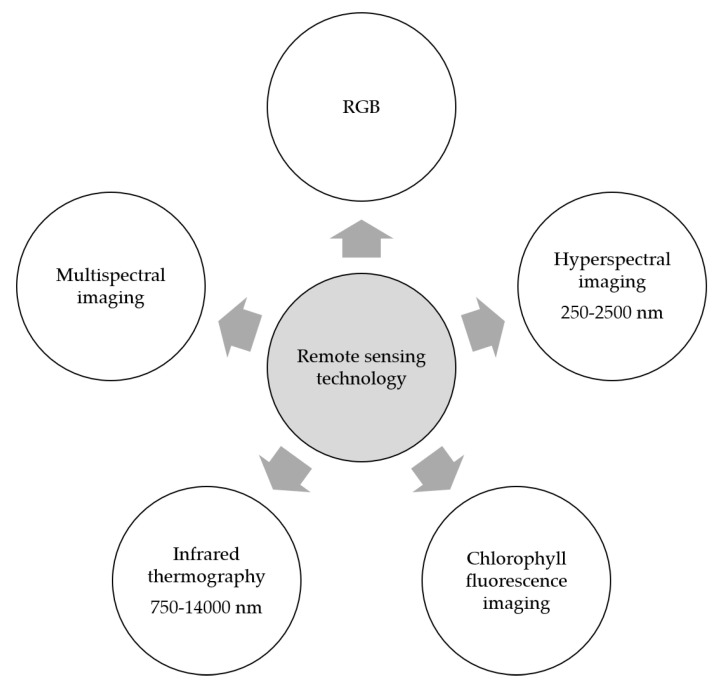

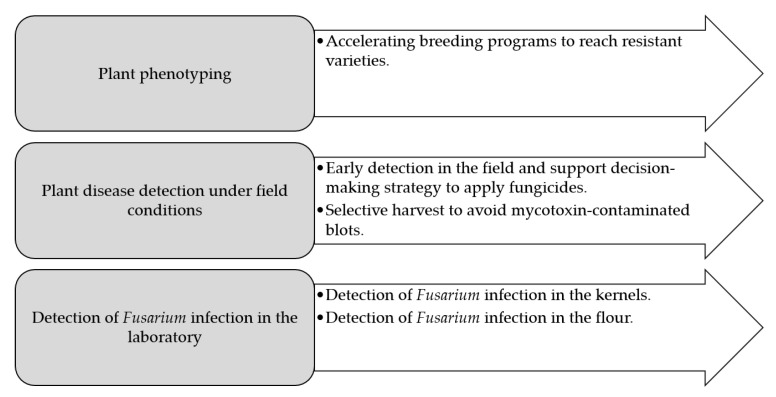
Spectral sensors and their application in integrated disease management of *Fusarium* head blight on wheat.

Optical sensors are among the remote sensing technologies that have been widely investigated in monitoring plant diseases as well as in plant phenotyping (Figure 4). These sensors include RGB imaging (red, green, and blue bands), multi- and hyperspectral imaging in the visible–near infrared range and the shortwave infrared range, infrared thermography in the spectral range 7500–14,000 nm and chlorophyll fluorescence imaging [13].

### 7.1. Spectral Techniques

Spectral sensors acquire the spectral reflectance of the object. Based on the number of recorded wavebands, these sensors are classified as multispectral sensors and hyperspectral sensors. Multispectral sensors record the spectral reflectance of individual wavebands (e.g., RGB wavebands or specific wavebands in the NIR range) [13], while hyperspectral sensors record the spectral reflectance over a wide number of wavebands in the electromagnetic spectrum from 250 to 2500 nm. This information is correlated to the plant pigments, chemical compounds, and the water content of the plant [11].

The main flaw of using hyperspectral imaging sensors in plant-disease detection is data complexity. To reduce data complexity, spectral vegetation indices (SVIs) can be derived from the spectral data based on a ratio between individual wavebands. Each of these indices can be used as an indicator of a specific compound of the plant which might be affected during the pathogenicity (e.g., chlorophyll, water content, or tissue structure). This data can be utilized in plant-disease detection using supervised or unsupervised machine learning methods. Reducing data complexity reduces calculation time and improves the accuracy of the machine learning approach in plant-disease detection [155].

Bauriegel et al. [156] investigated the feasibility of HSI in the VIS and NIR ranges for the early detection of FHB using data from controlled and field conditions. They showed that the best time for disease detection is at the beginning of the medium milk stage GS 71–85 according to the Lancashire scale [157]. Principal component analysis (PCA) was used to disclose the relevant wavelength of healthy and diseased wheat tissue. Based on this approach, the healthy and diseased areas of wheat spikes were correctly classified with an accuracy of 100 and 94%, respectively. A spectral angle mapper (SAM) was also able to classify the diseased area with an accuracy of 87%. However, the main drawback of SAM is that it is time-consuming. Another study showed the superiority of HSI in FHB detection under controlled conditions compared with the field conditions at the growth stage GS 71–73 [158]. Alisaac et al. [14] used SVM to discriminate healthy and *Fusarium*-infected wheat spikes based on the mean spectral signature and the SVIs derived from the mean spectral signature of the spikes. They reached an accuracy of >93% in the period 8–17 days after inoculation. In addition, it was possible to rank wheat varieties automatically according to their resistance to FHB using a non-metric multidimensional scaling approach based on the SVIs of the spikes [14]. Moreover, HSI showed promising results as a fast, non-invasive, and non-destructive method for pre-screening *Fusarium* infection and mycotoxin contamination on the kernel and flour scale. This can accelerate the kernel sorting procedure by replacing the laborious and cost-effective chemical methods [15,159].

Under field conditions, Ma et al. [160] used the spectral reflectance in the VIS, NIR, and SWIR ranges to detect FHB. Six feature bands correlated to FHB were extracted by continuous wavelet analysis (CWA). Afterward, these feature bands were utilized to establish a discrimination model using Fisher linear discriminant analysis (FLDA). They revealed an accuracy of 89% using this model. Jin et al. [161] used HSI in the VIS and NIR ranges to detect FHB on wheat under field conditions. They reached an accuracy of 85% using the neural network approach (NN). Color imaging was also used as an input for the deep neural network (DNN) approach to detect FHB on spike scale in the field. The accuracy of the model reached 92% at the milk stage of wheat [162]. Zhang et al. [163] integrated spectral and image data to detect FHB using the same approach with an accuracy of R^2^ = 0.97.

Unmanned aerial vehicles (UAVs) are an effective and flexible tool for acquiring high resolution images of a large acreage in a short time and with low costs. Zhang et al. [164] used UAV hyperspectral images for a quantitative detection of FHB in the field. They classified the field infection into mild, moderate and severe infection by fusing the spectral and image features acquired by UAV. FHB-monitoring models with accuracies of 98% and R^2^ = 0.88 were also developed based on UAV hyperspectral images to detect FHB under field conditions [165,166].

### 7.2. Infrared Thermography

Infrared thermography determines the plant temperature which reflects the water status of the plant. Plant pathogens influence the water balance in the plant tissue and this effect can be indirectly measured and visualized as a false-color image by IRT [13]. Maximum temperature difference (MTD) and average temperature difference (∆T) are parameters derived from IRT and can be successfully used in plant-disease detection. MTD represents the differences between the maximum and the minimum temperature within the object, while ∆T represents the difference between the average temperature of the ambient air and the average temperature of the object [167]. These parameters were implemented successfully using the support vector machine (SVM) approach to detect FHB on the spikelet scale [16].

### 7.3. Chlorophyll Fluorescence Imaging

Chlorophyll fluorescence imaging assesses the status of photosystem II (PSII) of the plant [168]. The basic fluorescence (F_0_) is the minimum value of fluorescence for dark-adapted PSII after excitation with low-intensity light but not enough for electron transport through PSII. The maximum fluorescence (Fm) is the maximum value of fluorescence for dark-adapted PSII after excitation with a saturating pulse. The variable fluorescence (Fv) represents the difference between Fm and F_0_, while the ratio Fv/Fm represents the maximum quantum yield of PSII photochemistry with a constant value of ≈0.83 for healthy plants [169]. FHB infection causes a significant reduction in the photosynthetic activity of wheat spikes; this reduction can be detected by CFI [16,170,171].

## 8. Future Perspectives

The damage caused by FHB includes yield losses and contamination of wheat kernels with mycotoxins. Therefore, applying fungicides at an early stage of pathogenicity should be considered in the management strategy of the disease [21]. This, in turn, needs an accurate control of disease incidence under field conditions. In addition, the selective harvest of healthy spikes could be an option to avoid infected spikes and reduces mycotoxin contamination [171]. This highlights the need for effective tools for real-time detection and identification of plant diseases in the field.

Former studies successfully implemented HSI and CFI to detect and discriminate healthy and FHB-infected spikes [156,170]. Mahlein et al. [16] showed the applicability of optical sensors, i.e., IRT, CFI, and HSI to detect FHB on the spikelet scale as early as three days after inoculation using a machine learning approach. In these terms, optical sensors are promising tool for future applications to support the decision-making strategy with FHB incidence to apply fungicides against FHB at an early stage of pathogenicity.

Based on the SVIs of wheat spikes, it was also possible to discriminate between healthy and FHB-infected spikes as well as spikes infected with different *Fusarium* species [14]. Using SVIs in FHB detection reduces the required data and time for FHB detection. This shows the feasibility of using multispectral instead of hyperspectral sensors for future real-time detection and identification of FHB under field conditions. This, in turn, gives the possibility of local treatment of infected spots in the field and reduces the quantities of plant protection chemicals. In addition, selective harvest aided by spectral sensors helps to avoid the infected spikes during harvest and reduces mycotoxin contamination of the harvested wheat kernels.

High yielding varieties with sufficient resistance to FHB are required to reduce yield losses and the use of plant protection chemicals substantially. Besides yield and disease resistance, different plant traits (e.g., plant height, lodging) have to be assessed simultaneously. from this point of view, using optical sensors, especially multi- and hyperspectral imaging sensors in phenotyping, can provide an objective assessment for plant traits in addition to reducing time and labor in the field [11].

Alisaac et al. [14] designed an automated model to rank wheat varieties according to their resistance to FHB based on multiple hyperspectral assessments of disease development during the pathogenicity [14,16]. This model needs to be optimized for future application under field condition.

Another factor to be considered in the breeding programs is kernel phenotyping for infection and mycotoxin contamination. This needs the quantification of *Fusarium* DNA and mycotoxin content in wheat kernels. However, the quantification methods are destructive, time-consuming, laborious, and expensive [172]. HSI proved the feasibility of detecting wheat kernels and flour contaminated with multiple levels of DON and fungal DNA of different *Fusarium* species [15]. Applying this tool allows the screening of a large number of wheat entries within a short time according to their DON and *Fusarium* DNA contents. Moreover, this technology will reduce the costs, labor, and time required for wheat kernel phenotyping against FHB.

## 9. Conclusions

In summary, combining different control measures has been shown to be an effective tool in the integrated disease management of FHB. These control measures must be considered before and after planting wheat. Before planting, applying suitable cultural practices and planting resistant varieties play a significant role in reducing disease incidence and severity. However, predicting and monitoring the disease will help in the decision making to apply biological and chemical control products during the growing season. In addition, selective harvesting by avoiding mycotoxin-contaminated plots is a useful tool to reduce mycotoxin contamination in harvested kernels. From this point of view, optical sensors, mainly IRT and HSI, are promising tools to monitor FHB infection on wheat.
